# Interfacial 2D Montmorillonite Nanocoatings Enable Sandwiched Polymer Nanocomposites to Exhibit Ultrahigh Capacitive Energy Storage Performance at Elevated Temperatures

**DOI:** 10.1002/advs.202204760

**Published:** 2022-10-30

**Authors:** Yifei Wang, Zongze Li, Thomas J. Moran, Luis A. Ortiz, Chao Wu, Antigoni C. Konstantinou, Hiep Nguyen, Jierui Zhou, Jindong Huo, Kerry Davis‐Amendola, Peinan Zhou, Bryan D. Huey, Yang Cao

**Affiliations:** ^1^ Electrical Insulation Research Center Institute of Materials Science University of Connecticut 97 N Eagleville Rd Storrs CT 06269 USA; ^2^ Department of Electrical and Computer Engineering University of Connecticut 371 Fairfield Way Storrs CT 06269 USA; ^3^ Department of Material Science and Engineering University of Connecticut 97 N Eagleville Rd Storrs CT 06269 USA

**Keywords:** 2D materials, dielectrics, energy storage, high temperature, interfaces

## Abstract

Polymer dielectrics are essential for advanced electrical and electronic power systems due to their ultrafast charge–discharge rate. However, a long‐standing challenge is to maintain their dielectric performance at high temperatures. Here, a layered barium titanate/polyamideimide nanocomposite reinforced with rationally designed interfaces is reported for high‐temperature high‐energy‐density dielectrics. Nanocoatings composed of 2D montmorillonite nanosheets with anisotropic conductivities are interposed at two kinds of macroscopic interfaces: 1) the interfaces between adjacent layers in the nanocomposites (inside) and 2) the interfaces between the surface of the nanocomposite and the electrode (outside). By revealing the charge transport behavior with Kelvin probe force microscope, surface potential decay, and finite element simulation, it is demonstrated that the outside nanocoatings are observed to diminish charge injection from the electrode, while the inside nanocoatings can suppress the kinetic energy of hot carriers by redirecting their transport. In this interface‐reinforced nanocomposite, an ultrahigh energy density of 2.48 J cm^−3^, as well as a remarkable charge–discharge efficiency >80%, is achieved at 200 °C, six times higher than that of the nanocomposite without interfacial nanocoatings. This research unveils a novel approach for the structural design of polymer nanocomposites based on engineered interfaces to achieve high‐efficient and high‐temperature capacitive energy storage.

## Graphical abstract

Layered polymer nanocomposites incorporating 2D montmorillonite nanocoatings at interfaces achieve significantly enhanced dielectric energy storage and breakdown performance. The outside nanocoatings are effective at blocking charge injection, while the inside ones redirect penetrating charges to dissipate in‐plane instead.

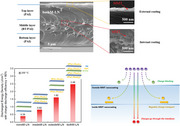

## 1 Introduction

Electrostatic capacitors are critical components for power electronics, power conditioning, and pulsed power systems, because of their high power density and superior capability of ultrafast charging–discharging.^[^ ^1^, ^2^, ^3^ ^]^ Compared with ceramic counterparts, polymer dielectrics possess inherent advantages of high voltage endurance, scalability, low cost, and high breakdown strength.^[^ ^4^, ^5^, ^6^, ^7^, ^8^, ^9^, ^10^, ^11^, ^12^ ^]^ However, they suffer from low operating temperatures and thus fall short of the demands for electrical energy storage and conversion in harsh environments.^[^ ^13^, ^14^ ^]^ For example, biaxially oriented polypropylene (BOPP), the state‐of‐the‐art commercially available polymer dielectric used in the power inverter, can only reliably operate at temperatures below 105 °C, while the engine compartment temperature in electric vehicles can exceed 120 °C.^[^ ^15^ ^]^ To leverage the benefits of BOPP based capacitors, cooling via cumbersome thermal management systems is therefore required—impacting the overall cost, maintenance, and reliability benefits.

To address this issue, a variety of polymers with favorable thermal stability and high glass transition temperatures have been examined for high‐temperature capacitive energy storage, such as polyimide (Kapton), fluorine polyester (FPE), polyetherimide (PEI), polycarbonate (PC), and poly(ether ether ketone) (PEEK).^[^ ^16^, ^17^ ^]^ Unfortunately, stable dielectric characteristics can only be preserved in those polymers at moderately high electric fields and up to their glass transition temperatures, while the high‐field/temperature electrical conduction cannot be effectively restricted. For example, the electrical conductivity of Kapton exponentially increases with temperature and electric field, which rises from 6.8 × 10^−13^ S m^−1^ at 50 MV m^−1^ to 3.5 × 10^−11^ S m^−1^ at 250 MV m^−1^ (at 150 °C).^[^ ^18^ ^]^ This not only leads to poor discharged energy densities and charge–discharge efficiencies but also generates Joule heating and ultimately thermal runaway, threatening the safe operation of the system.^[^ ^19^, ^20^ ^]^

The presence of surface states (due to chemical or physical defects) in polymers dramatically reduces the barrier height of the dielectric‐metal contact, and thus the intense charge injection at elevated temperatures and electric fields acts as the major conduction loss mechanism.^[^ ^21^, ^22^, ^23^, ^24^ ^]^ The emergence of ceramic nanocoatings in the last decade is recognized as a monumental achievement in the study of high‐performance polymer dielectrics, showing great promise for impeding charge injection. Chemical vapor deposition (CVD), as a relatively scalable method for the synthesis of 2D materials, has been carried out to manufacture insulating coatings such as boron nitride and silica (SiO_2_) on high‐temperature dielectrics.^[^ ^25^, ^26^ ^]^ However, compared with roll‐to‐roll scalable solution methods, the demanding processing conditions for CVD significantly limit the choices of coating materials and manufacturing throughput, not to mention the high cost. Moreover, it remains a major challenge for CVD to interpose 2D nanocoatings in the polymer bulk as charge transport barriers, e.g., introducing nanocoatings at the inside interfaces in layered polymer composites.

Here, we demonstrate a generic low‐cost and scalable approach that can modify inside and outside interfaces in layered nanocomposites with 2D nanocoatings. The nanocoatings composed of montmorillonite (MMT) nanosheets are interposed in layer‐structured barium titanate/polyamideimide (BT/PAI) nanocomposites via the combined layer‐by‐layer solution casting and spray coating processing. The benefits of the 2D nanocoatings are that they are electrically insulating out‐of‐plane to hinder charge injection from electrodes, while having relatively higher in‐plane ionic surface conductivity to redirect hot carriers for in‐plane dissipation instead of through‐film conduction.^[^ ^27^, ^28^, ^29^, ^30^ ^]^ This synergistic interfacial effect contributes to significantly suppressed conduction losses, allowing us to achieve an ultrahigh energy density of 2.48 J cm^−3^ and a charge–discharge efficiency >80% at 200 °C, which is six times higher than the nanocomposite without any interfacial reinforcements (0.39 J cm^−3^).

## 2 Results and Discussion

**Figure 1:**
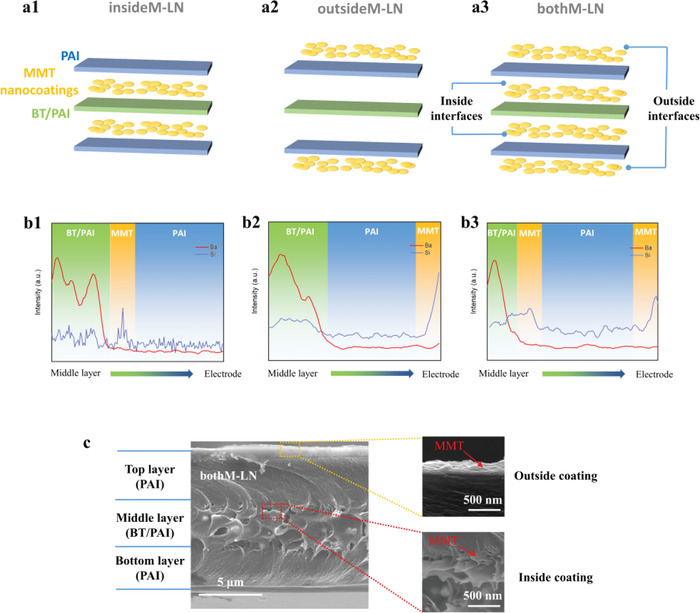
a) Schematic illustrations and b) energy dispersive X‐ray analysis line‐scans of the three engineered layered nanocomposites, revealing 2D MMT interfaces incorporated only at distinct regions of the overall film structures. c) Cross‐sectional SEM images of the optimized “bothM‐LN” specimen, which comprises MMT at every interface, as both inside and outside nanocoatings.

We designed and manufactured a library of BT/PAI layered nanocomposites (LN) with 2D MMT located 1) only at inside interfaces (insideM‐LN), 2) only at the outside surfaces (outsideM‐LN), and 3) both inside and outside (bothM‐LN). Their structures are illustrated in **Figure** **1**a and evidenced by line‐scan energy dispersive X‐ray analysis which clearly reveals MMT (identifiable by the Si prevalence) and BT (according to the presence of Ba) located as designed either at the interfaces or also in the film bulk, respectively (Figure 1b). These sandwiched nanocomposites consist of two outer PAI layers and a middle layer with 15 wt% BT nanoparticles, which can ensure both high breakdown strength and high permittivity. The high quality and flexibility of the composite films are demonstrated by scanning electron microscopy (SEM, Figure 1c) and optical images (Figure S1, Supporting Information).

**Figure 2:**
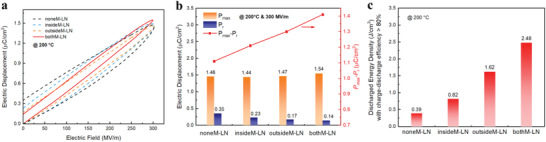
a) Unipolar D–E loops at 300 MV m^−1^ and 200 °C. b) Comparison of electric displacement at 300 MV m^−1^ and 200 °C. c) Comparison of the discharged energy density at above 80% charge–discharge efficiency.

To disclose the influence of the internally and externally interfacial reinforcement on the high‐field dielectric properties, electric displacement‐electric field (D–E) loops measurement was performed on the layered nanocomposites with and without 2D MMT coatings, as reflected in **Figure** **2**a. It is found that either inside or outside coatings can endow the layered nanocomposite apparently enhanced insulating capability, thus leading to slimmer D–E loops. For instance, lower values of remnant polarization can be found in insideM‐LN (0.23 µC cm^−2^) and outsideM‐LN (0.17 µC cm^−2^), compared with that in the nanocomposite without MMT nanocoatings (denoted as noneM‐LN, 0.35 µC cm^−2^), at an electric field of 300 MV m^−1^ and a high temperature of 200 °C (Figure 2b).

The most important thing to note is that bothM‐LN may inherit the favorable insulating capabilities of both inside and outside coatings, showing a much‐suppressed remnant polarization (*P*_r_ = 0.14 µC cm^−2^). Furthermore, owing to its two more interfaces containing higher contents of MMT with high dielectric permittivity, it is capable of possessing higher dielectric permittivity than either insideM‐LN or outsideM‐LN (Figure S3, Supporting Information), which also contributes to an enhanced polarization. For example, bothM‐LN delivers a maximum polarization (*P*_max_) of 1.54 µC cm^−2^ at 300 MV m^−1^, while that for insideM‐LN and outsideM‐LN are only 1.44 and 1.47 µC cm^−2^, respectively (Figure 2b). The concurrently improved *P*_max_ and suppressed *P_r_* lead to the highest difference value of *P*_max_ − *P*_r_ in bothM‐LN (1.41 µC cm^−2^), which suggests its favorable energy storage performance. High‐temperature (200 °C), high‐field (up to 450 MV m^−1^) capacitive energy storage properties were then studied by extracting charge–discharge efficiency and discharged energy density from D–E loops (Figures S4 and S6, Supporting Information). As expected, the discharged energy density for bothM‐LN at the charge–discharge efficiency >80% is 2.48 J cm^−3^, which is 536%, 202%, and 53% higher than that for noneM‐LN, inside‐LN, and outsideM‐LN, respectively (Figure 2c).

**Figure 3:**
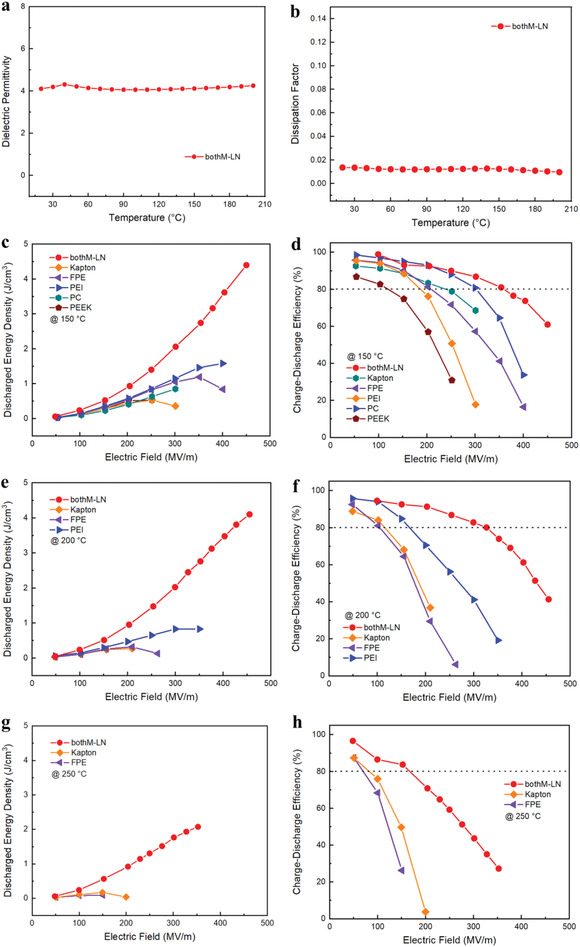
Temperature dependence of a) dielectric permittivity and b) dissipation factor for the optimal sample “bothM‐LN.” Discharged energy density (left column) and charge–discharge efficiency (right) of bothM‐LN and other conventional high‐temperature polymers at three distinct temperatures: c,d) 150 °C, e,f) 200 °C, and g,h) 250 °C.

To investigate the temperature dependent dielectric properties of bothM‐LN, the dielectric spectroscopy was carried out from room temperature up to 200 °C. Remarkably stable dielectric permittivity is seen in **Figure** **3**a, which shows a minor variation of less than 5% with a low dielectric loss of <2% (Figure 3b). Differential scanning calorimetry curves further prove the favorable high‐temperature resistance of bothM‐LN, where no apparent endo‐ or exo‐ thermal peaks can be observed (Figure S2, Supporting Information). We then studied the energy storage properties of these interfaces reinforced layered nanocomposites, over a temperature range from 150 to 250 °C (Figures S7 and S8, Supporting Information). As summarized in Figure 3c–h and Table S1 in the Supporting Information, bothM‐LN outperforms all high‐temperature polymer dielectrics (including Kapton, FPE, PEI, PC, and PEEK)^[^ ^25^, ^31^ ^]^ and recently reported high‐temperature polymer composites. For example, at 150°C, the busbar temperature in electric vehicle power train system, bothM‐LN can discharge ultrahigh energy density of 3.6 J cm^−3^ under 400 MV m^−1^, which is more than twice that of PEI (1.6 J cm^−3^), accompanied by a high efficiency of 73.7%, also more than twice that of PEI (33.8%). At 200 °C, only a minor decrease in energy density and efficiency can be observed in bothM‐LN. The advantages of bothM‐LN become more obvious when the temperature is raised to 250 °C. Most of the polymers cannot sustain under this harsh environment, and, moreover, the survivors (Kapton and FPE) can only operate at electric fields lower than 200 MV m^−1^. However, bothM‐LN can still deliver a high energy density of 2.1 J cm^−3^ at 350 MV m^−1^. Note that BOPP, as the benchmark material in the power converter/inverters in electric vehicles, can only discharge an energy density of ≈2 J cm^−3^ at a very low temperature of 85°C, with the protection of an additional cumbersome cooling system.

**Figure 4:**
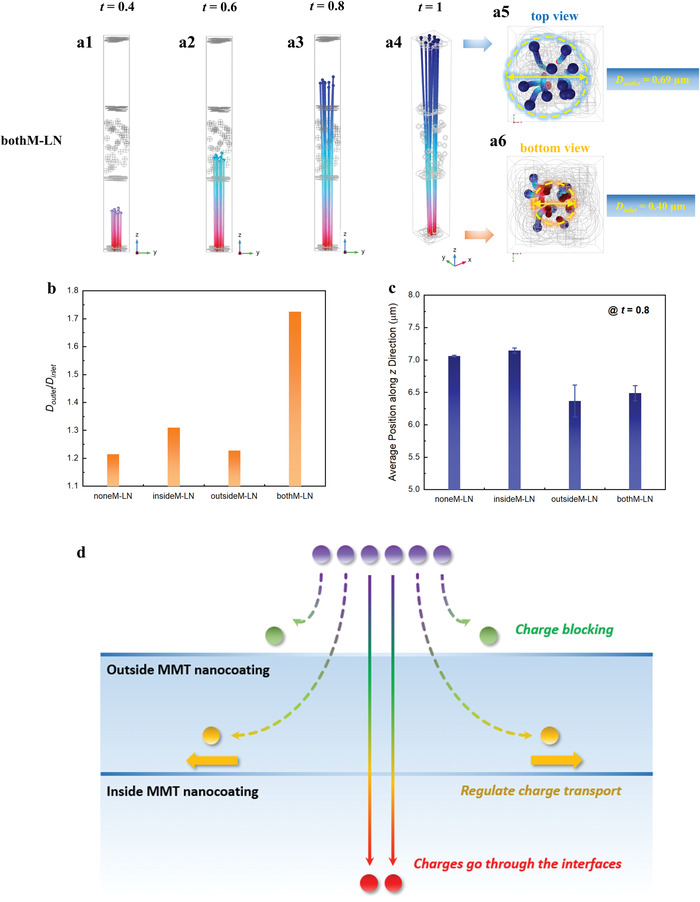
Finite element simulations. a) Representative charged particle trajectories in 3D models of the layered nanocomposites comprising 2D interfaces located both inside and outside. b) The ratio of outlet diameter to inlet diameter and c) the average depth (along *z*‐direction) of the charged particles at *t* = 0.8. d) Schematic illustration of the inside and outside interfacial effect, where the former mitigates charge injection from the electrode while the latter regulates any penetrating charges by preferential redirection and eventual dissiplane in‐plane.

The excellent dielectric performance of the interfaces reinforced nanocomposites is imparted by the substantial suppression of electrical conduction that would otherwise lead to high energy loss, degrade charge–discharge efficiency, and limit the practical energy density. To reveal the effect of the 2D MMT interfaces on the carrier transport process, we utilized the charged particle tracing module in COMSOL to simulate the time‐evolution position and velocity of charged particles in noneM‐LN, insideM‐LN, outside‐LN, and bothM‐LN. **Figure** **4**a and Figure S12 in the Supporting Information assume 3 keV electrons with respect to a grounded bottom surface, drifting under this high field until scattered by the engineered interfaces. It can be seen that the charged particles tend to diverge along the in‐plane direction (*xy* plane) in insideM‐LN, especially after they penetrate through the inside MMT nanocoatings, thus leading to an enlarged diffusion diameter. Figure S13a5,b5 presents the position of the charged particles in noneM‐LN and insidedM‐LN, respectively, when they arrive at the top high‐voltage electrode. The charged particles in insideM‐LN locate in a much larger circle area with a diameter of 0.54 µm than that in noneM‐LN (0.51 µm), owing to the hot carrier regulation effect of the inside MMT nanocoatings. On the other hand, the charged particles in outsideM‐LN fall behind that in noneM‐LN at every time stamp, indicating the blocking effect of the outside MMT nanocoatings on charge injection.

As bothM‐LN inherits the advantages of inside and outside nanocoatings from insideM‐LN and outsideM‐LN, simultaneously, the transport of charged particles is not only suppressed but also diverged. We adopted the ratio of outlet diameter to inlet diameter of the charged particles (*D*_outlet_/*D*_inlet_) to quantitatively describe the degree of particle divergence, and the average position of the charged particles (along *z*‐direction) at a specific time point (*t* = 0.8) was utilized to reflect their transport speed. As summarized in Figure 4b,c, the layered films with inside nanocoatings (insideM‐LN and bothM‐LN) have higher values of *D*_outlet_/*D*_inlet_, while the particles in the films reinforced by outside nanocoatings (outside‐LN and bothM‐LN) fall behind that in the other two counterparts. These results indicate that the outside nanocoatings are effective in blocking charge injection, while the inside ones redirect penetrating charges to dissipate in‐plane instead, as illustrated in Figure 4d. As the kinetic energy of the charged particles is low at the beginning stage (*t* < 0.1), the blocking effect of the outside nanocoatings is dominant, which directly alleviates the acceleration process of the charged particles. However, the charged particles would gain remarkable energy from the electric field force after they reach the middle of the nanocomposite (*t* ≈ 0.5). As a result, the inside nanocoatings cannot effectively block the high‐speed charged particles but disperse their kinetic energy along the in‐plane direction, owing to the anisotropic conductivity of 2D MMT. We adopt a ratio of the standard deviation to the average value to describe the discrete degree of the position and velocity of those charged particles (*ϕ*_p_ and *ϕ*_v_, respectively), as summarized in Figure S13 in the Supporting Information. As seen, the values of *ϕ*_p_ and *ϕ*_v_ in bothM‐LN are much higher than those in noneM‐LN. The introduction of 2D MMT results in the localized redistribution of the electric field (Figure S14, Supporting Information), which makes the charges motion paths more tortuous, thus leading to the suppressed conduction and energy loss (experimentally evidenced by the prebreakdown conduction measurement, Figure S9, Supporting Information).^[^ ^32^, ^33^ ^]^

**Figure 5:**
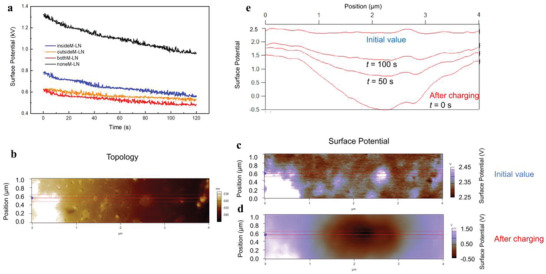
a) Surface potential decay of the layered nanocomposites with 2D interfaces after corona charging with a high voltage of 6 kV. b) Surface topography and surface potential from KPFM of the polymer with outside MMT coating c) before (initial value) and d) after charge deposition. e) Mean profiles of surface potential decay where indicated.

It has been demonstrated that charge accumulation and dissipation behavior can be inferred from potential measurements as a function of time and related to known field‐dependent mechanisms.^[^ ^34^ ^]^ We experimentally visualize such charge transport by corona charging the nanocomposite surface with a positive high voltage of 6 kV and then measuring the surface potential decay, as summarized in **Figure** **5**a. Compared with insideM‐LN, outsideM‐LN possesses an even lower initial surface potential, which confirms that the outside coating can more effectively prevent the charge injection. However, as time goes by, the surface potential decays faster in insideM‐LN. For example, the surface potential decreases from 0.78 to 0.56 kV after 120 s in insideM‐LN (28% reduction), which changes from 0.62 to 0.53 kV in outsideM‐LN (only 15% reduction). It is believed that the rapid charge dissipation in insideM‐LN is attributed to the internal MMT nanocoatings. BothM‐LN inherits the advantages of insideM‐LN and outsideM‐LN, where a low initial potential (0.62 kV) and a high charge dissipation rate (decreases to 0.48 kV, corresponding to a 23% reduction) can be achieved simultaneously.

The charge dissipation effect of MMT was further demonstrated at the nanoscale by probing the charge dynamics using Kelvin probe force microscopy (KPFM), following a procedure previously established for conventional as well as polymer nanocomposite dielectrics.^[^ ^35^, ^36^ ^]^ The topography and initial surface potential (Figures 5b–e) for the polymer with an outside MMT coating were first mapped for reference. The surface is next charged by applying a DC bias during point‐contact in the center of the field of view. Finally, the surface potential in this vicinity is repeatedly mapped every 25 s. The first such postcharging image demonstrates a uniform negative peak (Figure 5d). Cross‐sections from this and several later scans in the same location clearly reveal the charge dissipating. The magnitude of the potential for this MMT‐coated polymer rapidly decays to almost the initial state within 100 s, similar to the response to corona charging and discharging in Figure 5a. For comparison, equivalent experiments on pure BOPP revealed time constants for charges to dissipate to 1/*e* (36.8% of the deposited charge) as long as 1000 s or more.^[^ ^34^ ^]^ Moreover, there is little correlation between the mapped surface potential and the local microstructure, with smooth potential profiles, resulting from the effective charge distribution achieved by the engineered coating.

## 3 Conclusions

In summary, this work investigates the mechanisms for improved performance of nanocomposite polymeric dielectrics by engineering distinct interfacial architectures for 2D MMT at the inside and outside interfaces of BT/PAI nanocomposites. The results demonstrate that outside and inside nanocoatings are effective for blocking charge injection and redirecting hot carrier transport, respectively. Synergistically, this achieves a highly improved energy density of 2.48 J cm^−3^ and charge–discharge efficiency greater than 80% at 200°C when both inside and outside interfaces are engineered, outperforming the state‐of‐the‐art high‐temperature dielectric polymers and composites. Further modifications of the 2D nanocoatings, for instance by improving the insulating capacity along the through‐thickness direction and the conductivity along with in‐plane directions, can also be explored in the future for potentially even greater enhancements in the “barrier effects” reported already herein. By understanding and leveraging such interfacial effects for layered dielectrics, this work provides a pathway to explore advanced dielectrics with high energy density and charge–discharge efficiencies in extremely harsh environments.

## Conflict of Interest

The authors declare no conflict of interest.

## Supporting information

Supporting Information

## Data Availability

The data that support the findings of this study are available from the corresponding author upon reasonable request.
