# Supplementary material for: Interfacial 2D Montmorillonite Nanocoatings Enable Sandwiched Polymer Nanocomposites to Exhibit Ultrahigh Capacitive Energy Storage Performance at Elevated Temperatures
Source: Adv Sci (Weinh). 2022 Oct 30;9(35):2204760. doi: 10.1002/advs.202204760 (PMC9762287; doi:10.1002/advs.202204760)
Supplement: Supplementary file 1 — Supporting Information [file ADVS-9-2204760-s001.pdf]

ADVANCED SCIENCE
Open Access

Supporting Information

for *Adv. Sci.*, DOI 10.1002/advs.202204760

Interfacial 2D Montmorillonite Nanocoatings Enable Sandwiched Polymer Nanocomposites to Exhibit Ultrahigh Capacitive Energy Storage Performance at Elevated Temperatures

*Yifei Wang, Zongze Li, Thomas J. Moran, Luis A. Ortiz, Chao Wu, Antigoni C. Konstantinou, Hiep Nguyen, Jierui Zhou, Jindong Huo, Kerry Davis-Amendola, Peinan Zhou, Bryan D. Huey and Yang Cao**

Supporting Information

Interfacial 2D Montmorillonite Nanocoatings Enable Sandwiched Polymer Nanocomposites to Exhibit Ultrahigh Capacitive Energy Storage Performance at Elevated Temperatures

*Yifei Wang, Zongze Li, Thomas J. Moran, Luis A. Ortiz, Chao Wu, Antigoni C. Konstantinou, Hiep Nguyen, Jierui Zhou, Jindong Huo, Kerry Davis-Amendola, Peinan Zhou, Bryan D. Huey, and Yang Cao**

Dr. Y. Wang, Dr. Z. Li, Dr. C. Wu, A. C. Konstantinou, H. Nguyen, J. Zhou, J. Huo, K. Davis-Amendola, P. Zhou, Prof. Y. Cao

Electrical Insulation Research Center, Institute of Materials Science, University of Connecticut, 97 N Eagleville Rd, Storrs, CT, 06269, USA

Email: yang.cao@uconn.edu

Dr. Z. Li, J. Zhou, H. Nguyen, Prof. Y. Cao
Department of Electrical and Computer Engineering, University of Connecticut, 371 Fairfield Way, Storrs, CT, 06269, USA

Dr. T. J. Moran, L. A. Ortiz, K. Davis, P. Zhou, Prof. B. D. Huey

Department of Material Science and Engineering, University of Connecticut, 97 N Eagleville Rd, Storrs, CT, 06269, USA

Contents

Section 1. Supplementary text

Section 2. Structure characterization

Section 3. High-temperature dielectric characterization

Section 4. Finite element simulation

Section 5. Surface potential decay measurement

Section 1. Supplementary text

Sample Preparation: The interface reinforced layered nanocomposites were manufactured by combined technology of doctor blade casting and spray coating. Taking the preparation of bothM-LN as an example, PAI resin (Tritherm A981-H-25) was diluted by dimethylformamide (DMF, Fisher Chemical) to make the precursor solution of the top and the bottom layer. For the middle layer, the precursor was prepared by dispersing BT nanoparticles (100 nm, Acros Organic) in a mixed solution of PAI and DMF by tip-type ultrasonication. Montmorillonite (MMT) nanosheets were dispersed in DMF to make the spraying solution, in which PAI resin was also dissolved as the binder of MMT. The weight ratio of MMT and PAI is 1:4, while the content of MMT+PAI is 4 wt% in the solution.

To make the layered composite film, a nanometric layer of MMT was sprayed on the glass substrate as the outside interface, followed by casting a micrometric layer of PAI. After it is dried at 80°C for 10 min, the casting and spraying processes were carried out in turn until all layers and interfaces were stacked up completely. All 2D interfaces were manufactured from the same precursor with a fixed MMT content. Then, the film was completely dried at 130°C overnight and transferred to a vacuum oven for annealing at 300°C for 2h. The insideM-LN and outsideM-LN were prepared by the same process by eliminating some of the steps that are not needed corresponding to their respective structures. The layered composite films with different numbers of layers and the amount of montmorillonite (MMT) were also manufactured to reveal their influence on the dielectric properties.

Characterization: The morphologies were characterized by scanning electron microscopy (SEM) combined with energy dispersive x-ray analysis (EDAX) (FEI Teneo Verios) to verify the micro-structures. Differential scanning calorimetry (DSC) was conducted using a TA Instruments Q-100. Gold/palladium (Au/Pd) electrodes were sputtered on the layered films (6002-8 Ted Pella, Inc.) for electrical measurements. Dielectric spectra were obtained over wide temperature and frequency ranges using an impedance analyzer (Solartron 1260) and a resistance meter. The high field electric

displacement-electric field (*D*-*E*) loops were measured at 100 Hz with a modified Sawyer-Tower circuit. For the surface potential decay test, a pair of needle-to-plate electrodes were used, where the sample was placed on the plate electrode while 6 mm away from the needle electrode. A DC voltage of 6 kV was applied to the needle to perform the corona charge for 1 min, then the sample was transferred under a probe to monitor the surface potential decay for 2 min. (Figure S15) Prebreakdown conduction at elevated temperature was carried out with a lab-designed capacitive cancellation measurement system. The capacitive current can be successfully canceled by a dynamic gain controlled negative feedback loop, and the signal output reflects the time-integrated conduction current.

Finite Element Simulation: 3D models of the layered nanocomposites with a size of 1×1×12.4 μm^3 were constructed in COMSOL, where nanoparticles and nanosheets were incorporated, located at the middle layer and the interfaces, respectively. The top surfaces of the models are endowed with a high voltage of 3 kV and the bottom surfaces are grounded. The dielectric permittivities of PAI, BT, and MMT were set for 4.5, 2000, and 500, respectively. The conductivities of PAI and BT were 10^{-15} S m^{-1} and 10^{-11} S m^{-1}, respectively. To mimic the anisotropic electrical conductivities of MMT nanosheets, a high surface conductivity of 10^{-3} S m^{-1} and relatively low bulk conductivity of 10^{-8} S m^{-1} were applied.[1,2] The electric field was simulated first, which was adopted as the electric force ($\boldsymbol{F_t}$) to drive the movement of the charged particles in the Charged Particle Tracing (CPT) Module.

$$F_t = \frac{d(m_p v)}{dt} = ZeE$$

$$E = -\nabla V$$

where

m_p – particle mass

$\boldsymbol{v}$ – particle velocity

t – time

e – elementary charge = 1.60×10^{-19} C

Z – particle charge

E – electric field

V – electric potential

Ten charged particles, that were assumed as electrons with charges of -1.60×10^{-19} C and masses of 9.11×10^{-31} Kg, were injected from a circle region on the bottom surface with a diameter of ~0.4 μm, and accelerated by the electric force along the z-direction. Time-dependent particle trajectories can be simulated with a specified time range.

KPFM: Kelvin Probe Force Microscopy (KPFM),[3,4] an extension of Atomic Force Microscopy (AFM), was applied to investigate the dissipation of surface charges for nanocomposite and control dielectrics. This is based on a dual-pass procedure with a commercial AFM (Asylum Research Cypher-ES), where AC-mode AFM is first employed in order to track the surface morphology. Next, non-contact measurements are performed at a fixed tip-sample separation (40 nm), allowing detection of electric field force gradients induced by any charges beneath the conducting tip (AppNano ANSCM-PA). Technically, KPFM maps tip biases necessary to null the capacitive forces acting between the tip and sample, which only occurs when their potentials are equal and can be maintained via a feedback loop. Multiple images of the surface potential distribution are acquired as a function of known time after an initial charging event at a fixed location. The pattern, magnitude, and even time constants for the charge decay can thereby be analyzed as already reported for nanocomposite[5] as well as conventional ceramic dielectrics[6].

Section 2. Structure characterization

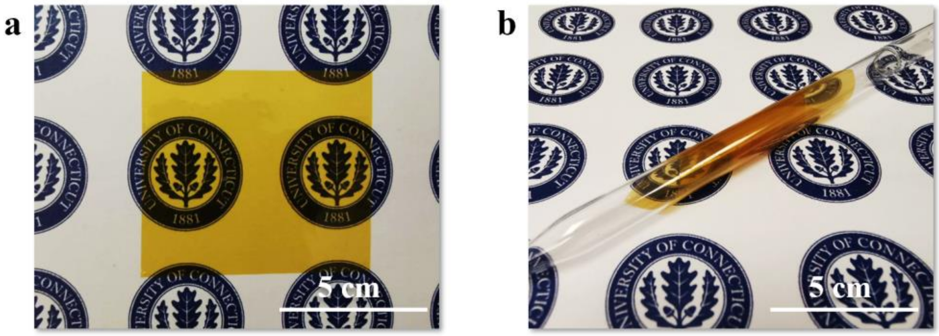

Figure S1. Photographs of the BT/PAI layered polymer nanocomposite reinforced by 2D MMT interfaces, which are a) laid flat on a paper and b) wrapped on a glass tube.

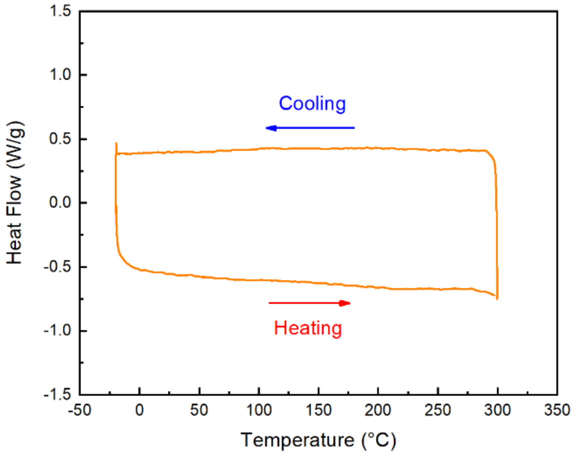

Figure S2. DSC curves of bothM-LN.

Section 3. High-temperature dielectric characterization

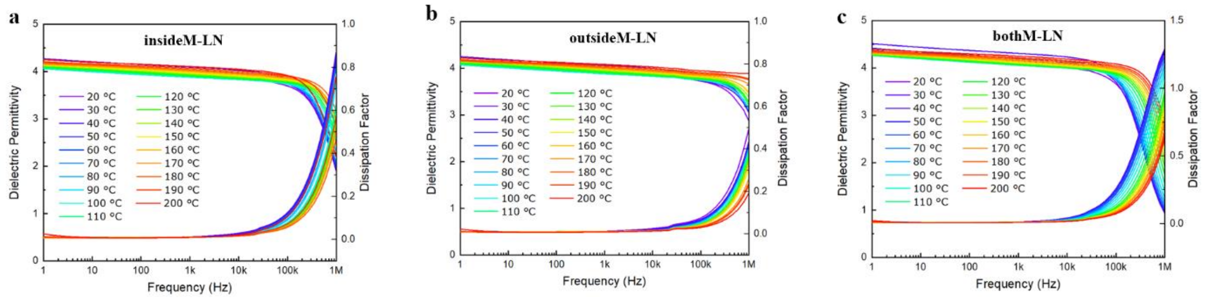

Figure S3. Frequency-dependent dielectric permittivity and dissipation factor of a) insideM-LN, b) outsideM-LN, and c) bothM-LN measured from room temperature to 200℃. As both insideM-LN and outside-LN have two interfacial nanocoatings with the same MMT content, no recognizable difference can be seen in dielectric permittivity.

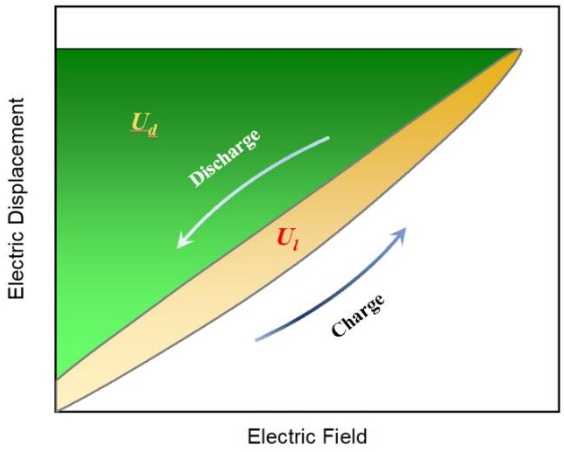

Figure S4. Schematic *D*-*E* loops of a dielectric material. The areas of yellow and green regions denote the value of energy loss (U_l) and discharged energy density (U_d), respectively. The charge-discharge efficiency is defined by the ratio of U_d to U_l+U_d.

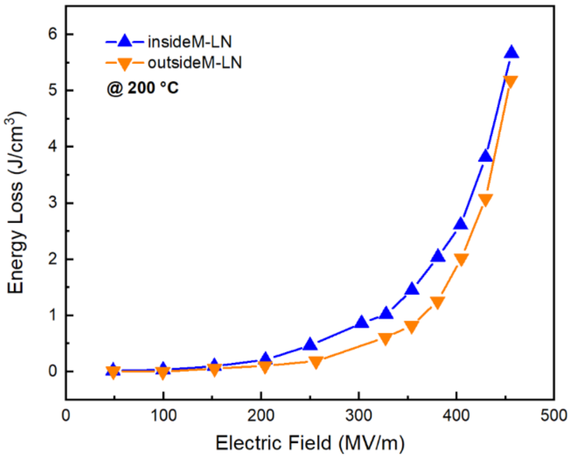

Figure S5. Electric field dependent energy loss of insideM-LN and outsideM-LN at 200°C. It is found that the outside coating can endow the nanocomposites with a better insulating capability compared with the inside coating. As seen, the energy loss decreases from 0.83 J cm^{-3} for insideM-LN to 0.44 J cm^{-3} for outsideM-LN at 300 MV m^{-1}.

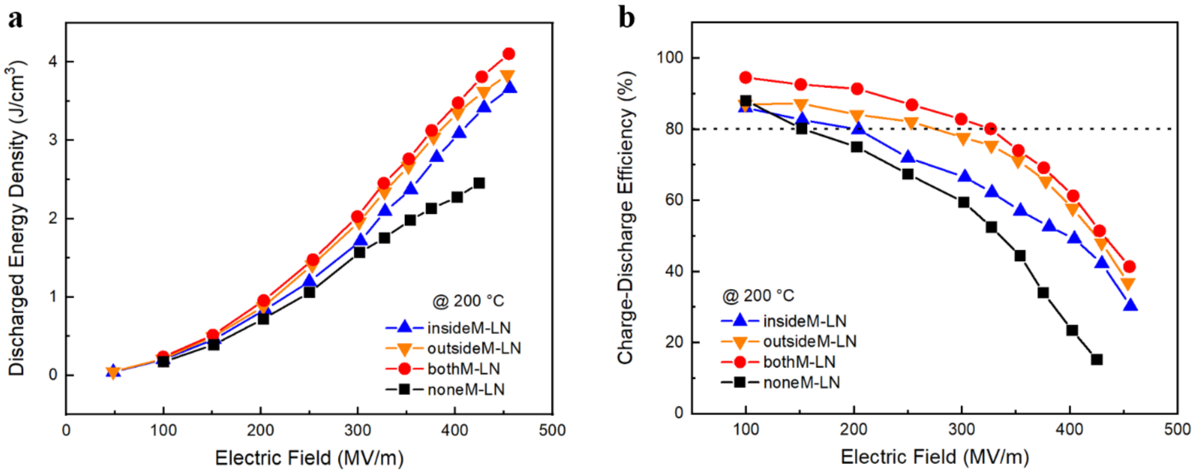

Figure S6. Electric field dependent a) discharged energy density and b) charge-discharge efficiency of insideM-LN, outsideM-LN, bothM-LN, and noneM-LN at 200°C.

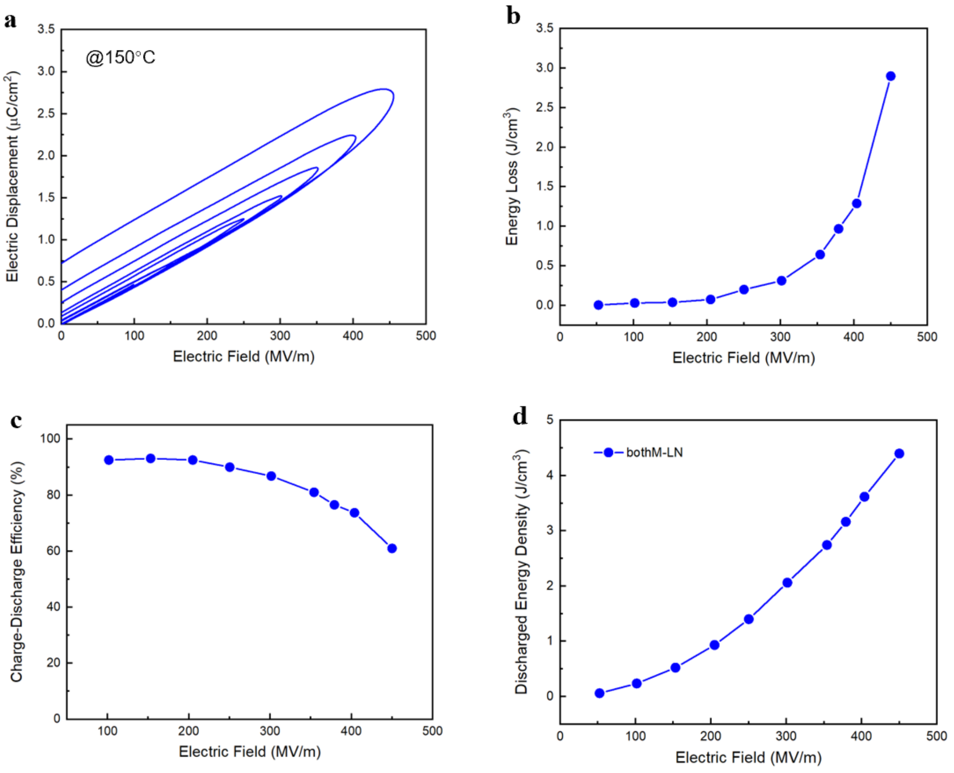

Figure S7. *D-E* loops, energy loss, charge-discharge efficiency, and discharged energy density of bothM-LN at 150°C.

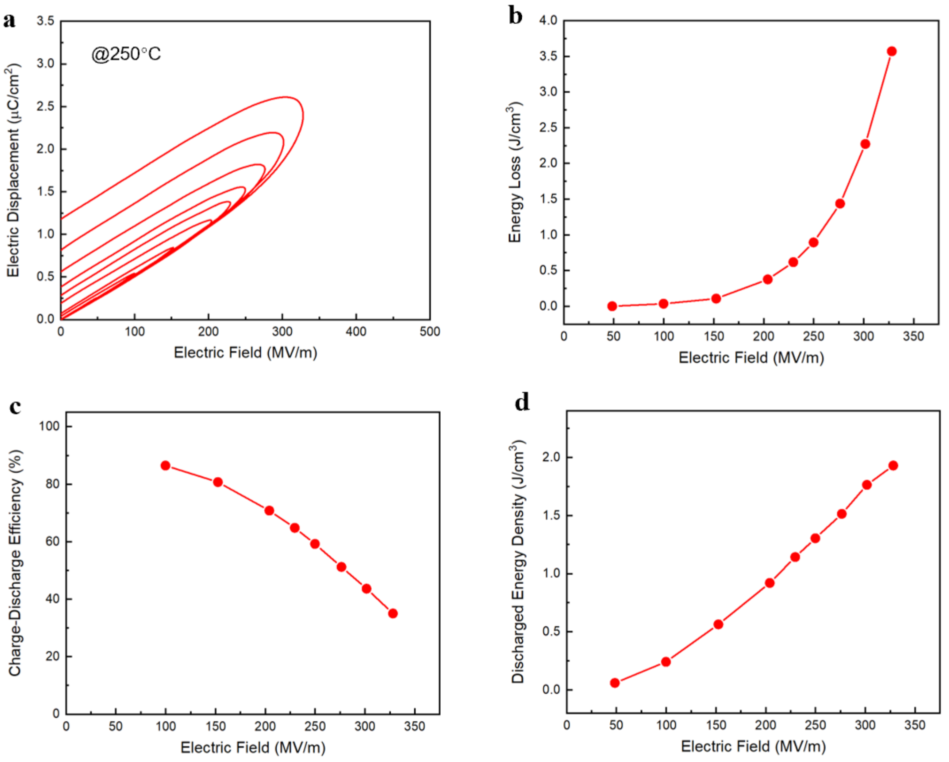

Figure S8. *D-E* loops, energy loss, charge-discharge efficiency, and discharged energy density of bothM-LN at 250°C.

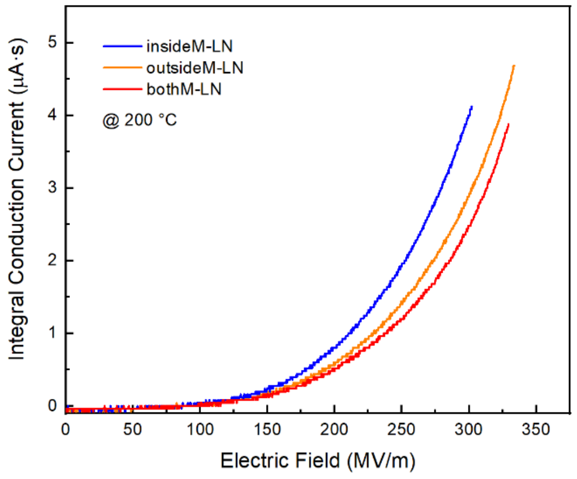

Figure S9. Integral conduction currents with the increment of the electric field in insideM-LN, outsideM-LN, and bothM-LN at 200°C. The onset points for high field conduction can be seen in all samples at ~ 200 MV m^{-1}, which indicates a rapid increase of conduction. The upward shift of the onset point from insideM-LN to bothM-LN suggests the enhanced electrical insulation in the layered nanocomposites with both inside and outside MMT nanocoatings, which coincides with its improved charge-discharge efficiency.

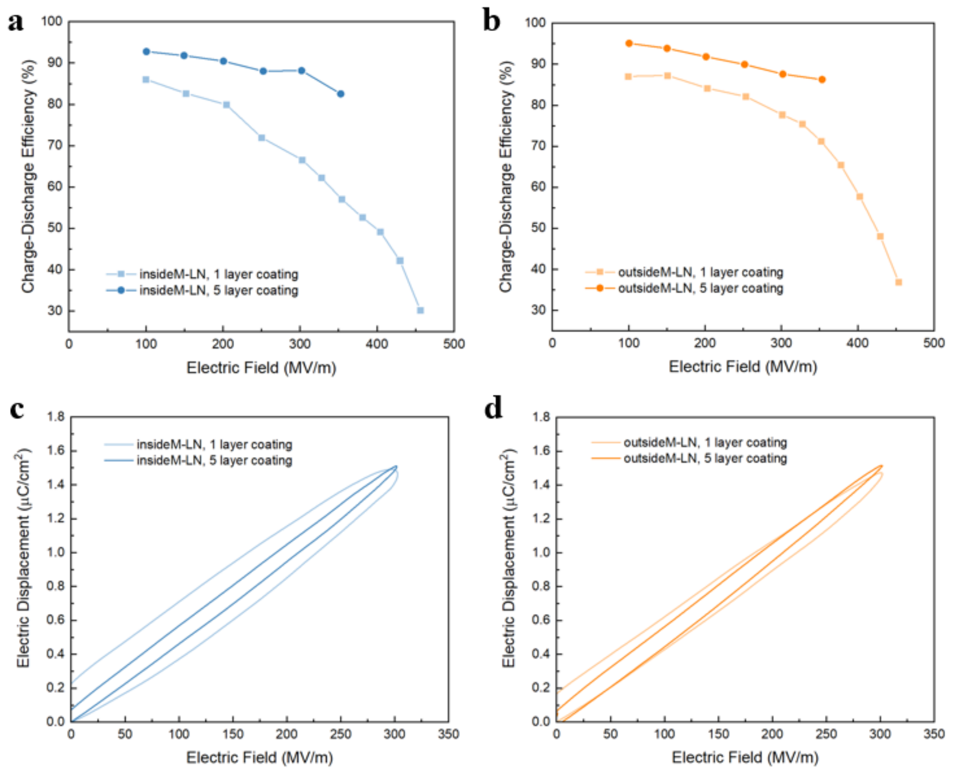

Figure S10. Discharged energy density of (a) insideM-LN and (b) outside-LN with different coating thicknesses at 200 °C. *D*-*E* loops of (a) insideM-LN and (b) outside-LN with different coating thicknesses at 300 MV m^{-1} and 200 °C.

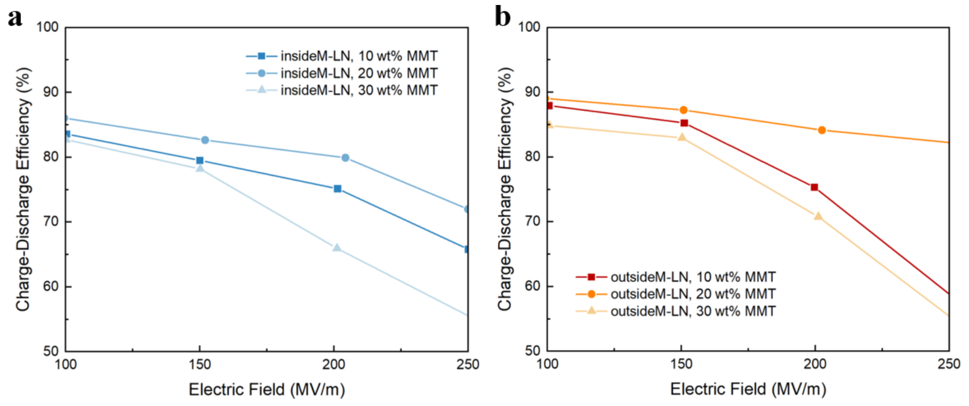

Figure S11. Discharged energy density of (a) insideM-LN and (b) outside-LN with different MMT contents at 200 °C.

Table S1. Discharged energy density at efficiency > 80% of bothM-LN and recently reported high-temperature polymer composites.

	Discharged Energy Density at Efficency > 80%	
	200 °C	250 °C
**bothM-LN (this work)**	**2.48**	**0.75**
c-BCB/Al_2O_3 NPLs[5]	2.4	-
c-BCB/BN[6]	1.6	0.7
A-P-A-P-A[7]	2.3	-
BN-coated PEI[8]	1.25	-

c-BCB: crosslinked divinyltetramethyldisiloxane-bis(benzocyclobutene); NPLs: nanoplatelets; BN: boron nitride; A-P-A-P-A: Al_2O_3-PI- Al_2O_3-PI- Al_2O_3 laminated film

Section 4. Finite element simulation

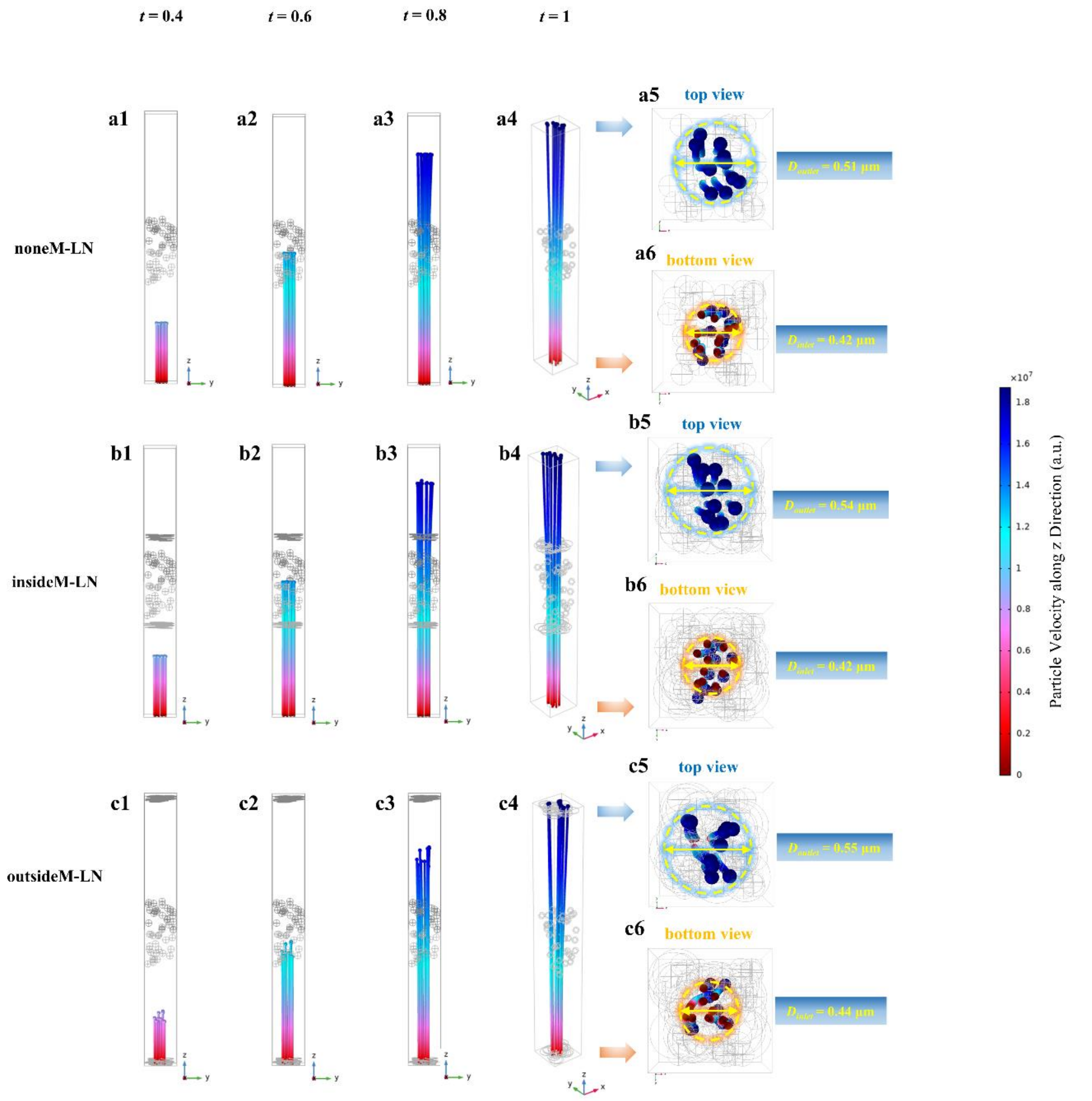

Figure S12. Charged particle trajectory in 3D models of a) noneM-LN, b) insideM-LN, and c) outsideM-LN, simulated by finite element method.

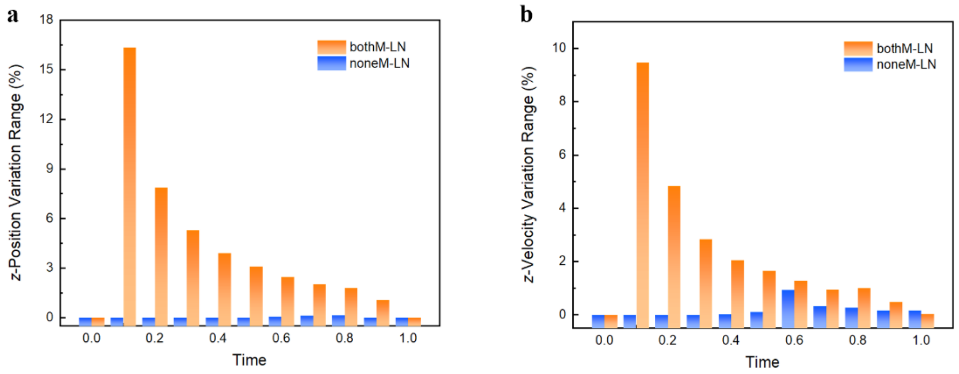

Figure S13. a) position and b) velocity variation range of the charged particles in the model of bothM-LN (denoted as ϕ_p and ϕ_v, respectively). The discrete degree is calculated by a ratio of the standard deviation to the average value.

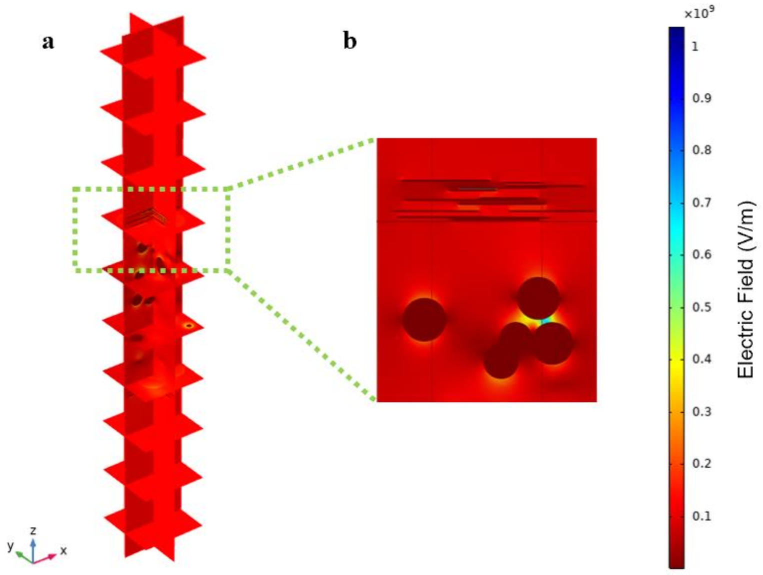

Figure S14. a) electric field distribution in bothM-LN, simulated by finite element method. b) localized electric field distribution, which shows the distorted electric field around BT and MMT nanofillers.

Section 5. Surface Potential Decay Measurement

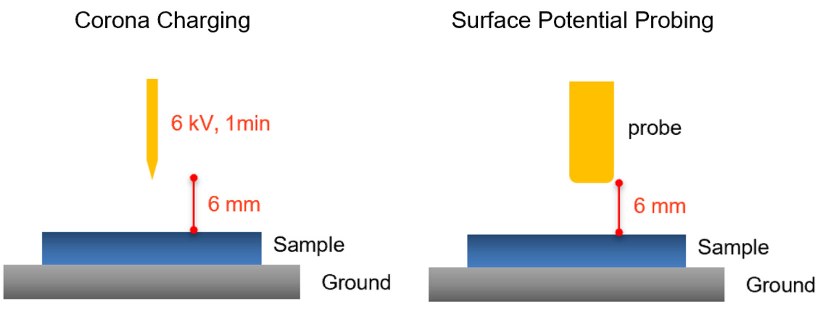

Figure S15. Schematic setup of surface potential decay measurement.

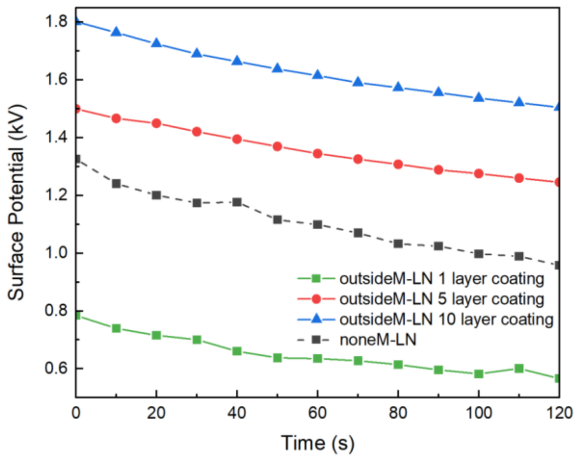

Figure S16. Surface potential decay of outside-LN with different coating thicknesses.

References

[1] A. A. Guseinov, *Izvestiya, Physics of the Solid Earth* **2017**, *53*, 845.

[2] J. Tokarský, L. Kulhánková, V. Stýskala, K. Mamulová Kutláková, L. Neuwirthová, V. Matějka, P. Čapková, *Applied Clay Science* **2013**, *80-81*, 126.

[3] M. Nonnenmacher, M. o'Boyle, H. K. Wickramasinghe, *Appl. Phys. Lett.* **1991**, *58*, 2921.

[4] M. Zhao, V. Sharma, H. Wei, R. R. Birge, J. A. Stuart, F. Papadimitrakopoulos, B. D. Huey, *Nanotechnology* **2008**, *19*, 235704.

[5] B. Zhang, J. Liu, M. Ren, C. Wu, T. J. Moran, S. Zeng, S. E. Chavez, Z. Hou, Z. Li, A. M. LaChance, T. R. Jow, B. D. Huey, Y. Cao, L. Sun, *Adv. Mater.* **2021**, *33*, e2101374.

[6] T. J. Moran, K. Suzuki, T. Hosokura, A. Khaetskii, B. D. Huey, *J. Am. Ceram. Soc.* **2021**, *104*, 5157.
